# Reversible dementia: subclinical seizure in early‐onset dementia

**DOI:** 10.1002/ccr3.843

**Published:** 2017-02-06

**Authors:** Elysia Tjong, William McHugh, Yen‐Yi Peng

**Affiliations:** ^1^ Department of NeurologyRenown Institute for Neurosciences, Renown HealthUniversity of NevadaRenoNevadaUSA

**Keywords:** Amyloid beta, Aß (_1‐42_)/t‐tau index, early‐onset alzheimer's disease, reversible dementia, subclinical seizure, tau

## Abstract

We report a case of early‐onset dementia with subclinical seizures. Aggressive seizure control improved the patient's cognition. Commonly, an EEG is only performed following overt behavioral seizures. Therefore, subclinical seizures tend to be underdiagnosed. Serial or extended EEG should be seriously considered in patients with early‐onset dementia.

## Introduction

Subclinical seizures are subtle/clinically unrecognized seizures apparent only on EEGs, which denotes focal seizures without impairment of consciousness. Furthermore, there are no observable motor or autonomic components. Patients may or may not have subjective sensory or psychic phenomena 1, 2. Often, EEG is not deemed as part of routine assessments in patients being evaluated for dementia without clinically evident epileptic seizures 3, 4. Therefore, subclinical seizures tend to be underdiagnosed 3.

Epileptic activity is known to be more prevalent in patients with dementia, and seizures are more common in patients with early‐onset dementia, aged between 50 and 60 years 5. Cognitive decline secondary to epilepsy may be reversible. It is imperative to identify reversible dementias, even if partial or full reversal of the cognitive symptoms cannot be guaranteed 6.

## Case Report

We present a case of a 61‐year‐old woman with progressive cognitive decline for about 4 years. She reported losing the ability to handle her profession. She also reported having trouble with word finding and short‐term memory problems.

Since the onset of symptoms, she was evaluated by neurologists who specialize in dementia including a separate referral to the memory and aging center of a tertiary medical center for an extensive workup. Two years after onset, the MMSE score was 27 of 30 (recall 0/3) documented in one office visit. Three years after onset, the MMSE score was 26/30 (recall 0/3 and missed county) documented in another outpatient evaluation. After these evaluations, the patient was advised not to drive. Her symptoms met the criteria set by National Institute of Neurological and Communicative Disorders for probable AD. CSF Aß (_1‐42_) beta amyloid protein was 359.9 pg/mL (low Aß (_1‐42_) beta amyloid protein <1240 pg/mL), t‐tau was 743.9 pg/mL (high tau >420 pg/mL), p‐tau was 105.5 pg/mL, and Aß (_1‐42_)/t‐tau index [ATI, calculated as Aß (_1‐42_)/(240 + 1.18 t‐tau)] was 0.32. (reference ranges: abnormal (i.e., AD range) values were ATI < 1.0 and p‐tau > 61 pg/mL) 7. The combination of high tau (i.e., >420 pg/mL) and low Aß (_1‐42_) beta amyloid protein (i.e., <1240 pg/mL) has a sensitivity of greater than 95% in demented >60‐year‐old patients and a specificity of greater than 60% (Athena Neuroscience Inc., Worcester, MA, ADmark™ Assays) 8. The above CSF studies performed in Athena Diagnostics were consistent with AD. Serum microsomal – thyroperoxidase (TPO) Abs, CSF oligoclonal bands, CSF Tropheryma whipplei polymerase chain reaction (PCR), and CSF antibodies in the complete Encephalopathy‐Autoimmune Panel of Mayo Clinic Laboratories were all negative, which include NMDA‐R Ab CBA, VGKC‐complex Ab IPA, GABA‐B‐R Ab CBA, AMPA‐R Ab CBA, ANNA‐1 (antineuronal nuclear Ab, type 1), ANNA‐2 (antineuronal nuclear Ab, type 2), ANNA‐3 (antineuronal nuclear Ab, type 3), AGNA‐1 (antiglial nuclear Ab, type 1), PCA‐1 (purkinje cell cytoplasmic Ab type 1), PCA‐2 (Purkinje cell cytoplasmic Ab type 2), PCA‐Tr (Purkinje cell cytoplasmic Ab type Tr), amphiphysin Ab, CRMP‐5‐IgG negative titer <1:2 (collapsin response mediator protein 5) and GAD‐65 antibody.

The patient was treated as AD accordingly (donepezil 23 mg once daily). EEG was not offered in the first 4 years of disease onset as the patient never reported clinically evident spells or epileptic seizures. Prior to EEG testing, the patient was deemed to be a candidate for assisted living.

**Figure 1:**
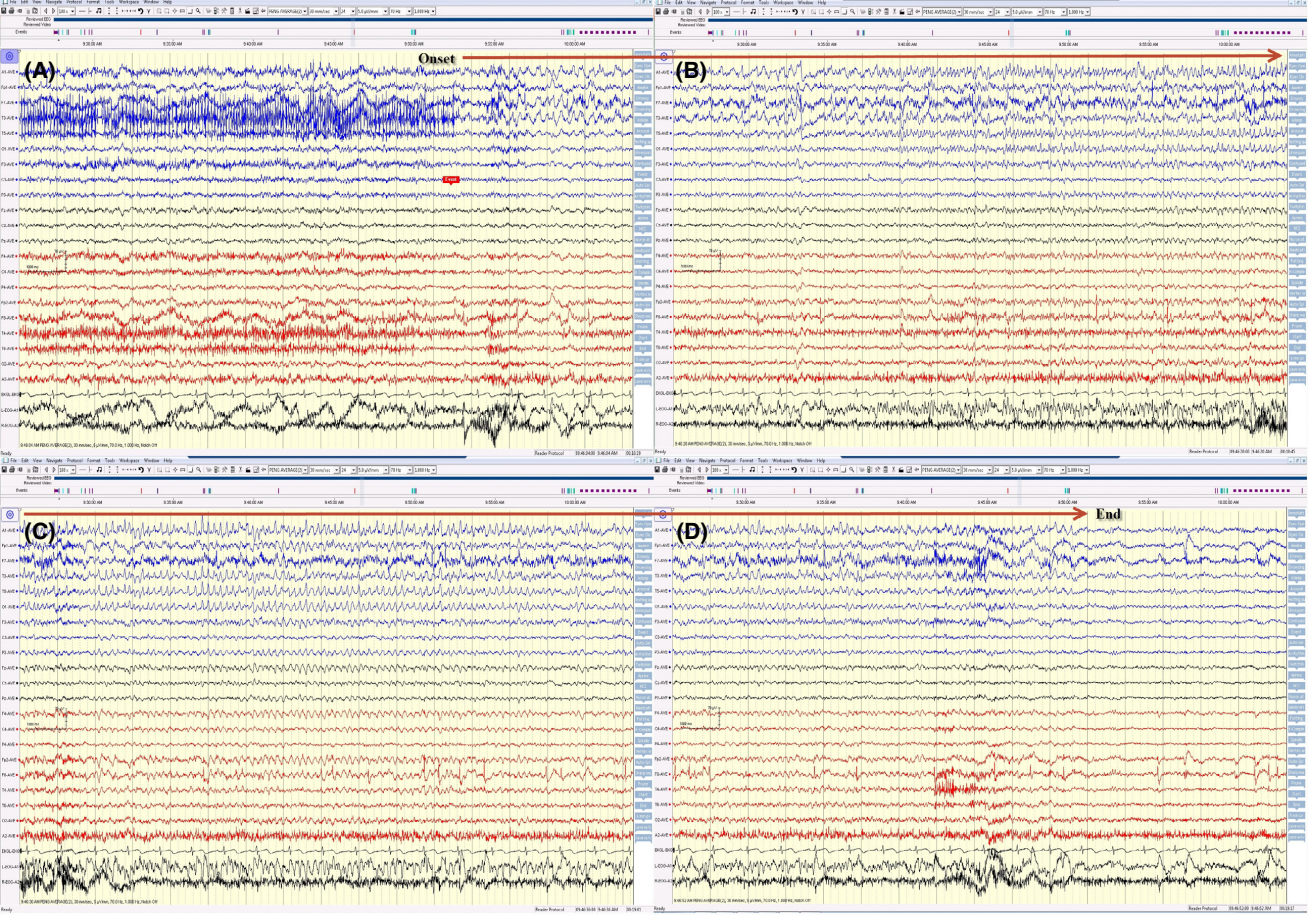
(A, B, C, and D) This electrical seizure started with sudden appearance of rhythmic 4 Hz activity (maximum at F7, T3) and evolution in amplitude, frequency, and morphology of this rhythmic activity spread over the left hemisphere displayed on an average reference montage. This electrical seizure lasted for around 1 min long.

**Figure 2:**
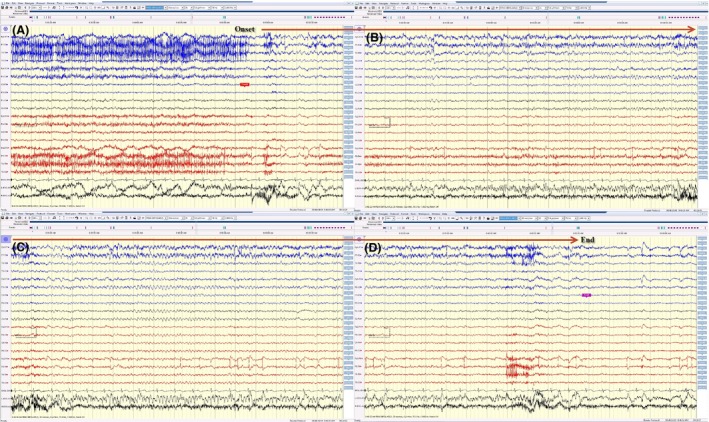
(A, B, C, and D) The same electrical seizure in Figure 1 displayed on a bipolar montage. This electrographic seizure could be easily missed on routine bipolar montage because of extensive in‐phase cancellation.

Early onset of cognitive deficits motivated us to be extensive in diagnostic workup 9, 10, 11, 12, 13. The Scalp EEG documented a one‐minute long subclinical seizure with its focus originating from the left temporal region. (Figs 1 and 2). This electrographic seizure could be missed on routine bipolar montage because of in‐phase cancellation. (Fig. 2) Levetiracetam (250–500 mg twice a day) was added but the patient's family did not notice any cognitive improvement. She had been taking 500 mg daily valproic acid for psychiatric disorders (anxiety and depression) for 1 year at the time of the routine EEG test.

**Figure 3:**
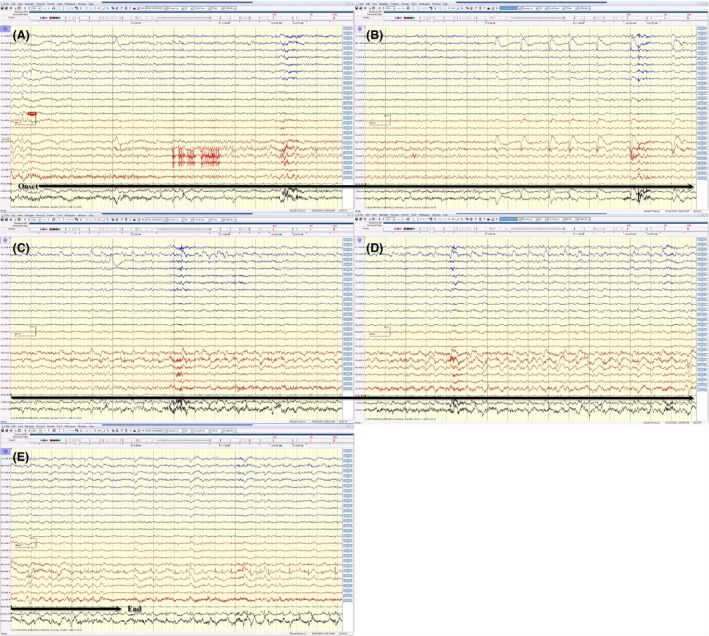
(A, B, C, D and E) This electrical seizure started with sudden appearance of sharp activity (maximum at FP2, F8, T4), followed by rhythmic activity, with evolution in amplitude, frequency, and morphology of this rhythmic activity spread over the right temporal region displayed on an average reference montage. This electrical seizure lasted for around 70 sec.

**Figure 4:**
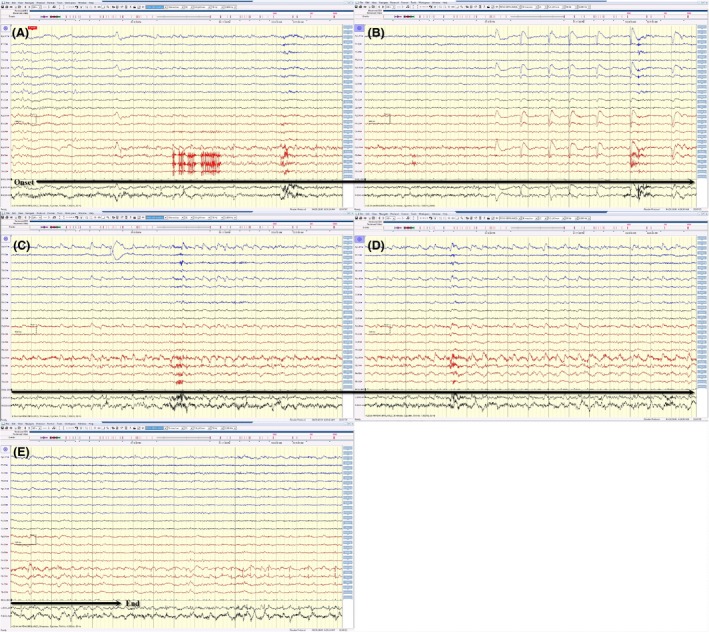
(A, B, C, D, and E) The same electrical seizure in Figure 3 displayed on a bipolar montage. This electrographic seizure became less conspicuous on routine bipolar montage because of extensive in‐phase cancellation.

Extended video EEG was subsequently performed to guide future treatment. Both valproic acid and levetiracetam were held on the first day of video EEG monitoring. On the first day, the video EEG was not remarkable and the patient remained oriented, with the ability to recall three of three words after 5 min. On the second day, the patient developed four subclinical seizures lasting from 30 sec to 1 min with the seizure originating from the right temporal region. On the third day, the patient developed clusters of electrographic seizures approximately once per hour. Each electrographic seizure lasted from a minute to one and a half minutes. (Figs 3 and 4). There was no impairment of consciousness. There were no observable motor or autonomic components either. These electrographic seizures could be missed on routine bipolar montage because of in‐phase cancellation. (Fig. 4) The majority of the seizures recorded during this admission had their seizure focus from the right temporal lobe. Only a couple of them had their focus from the left temporal region like that of the outpatient routine EEG. She remained oriented on day three of video EEG. Upon further request, she was able to recognize that the onset of right temporal seizures coincided with peculiar feelings in her stomach twice. She was not able to associate any feelings with those seizures from the left temporal region. On the fourth day, her amnesia became obvious. She was not able to recall that she had those sensory seizures. Nor could she recall the conversation with us the day before. She took the EEG leads off since she was confused. Epileptic amnesia secondary to prolonged postictal cognitive deficit was likely.

Levetiracetam was reinstituted and its dose was increased (levetiracetam 2000 mg once and followed by levetiracetam 750 mg three times a day). Her amnesia and confusion were partially alleviated the next day. This extended video EEG confirmed that subclinical seizures contribute to the patient's cognitive deficits. Aggressive seizure control is warranted.

**Figure 5:**
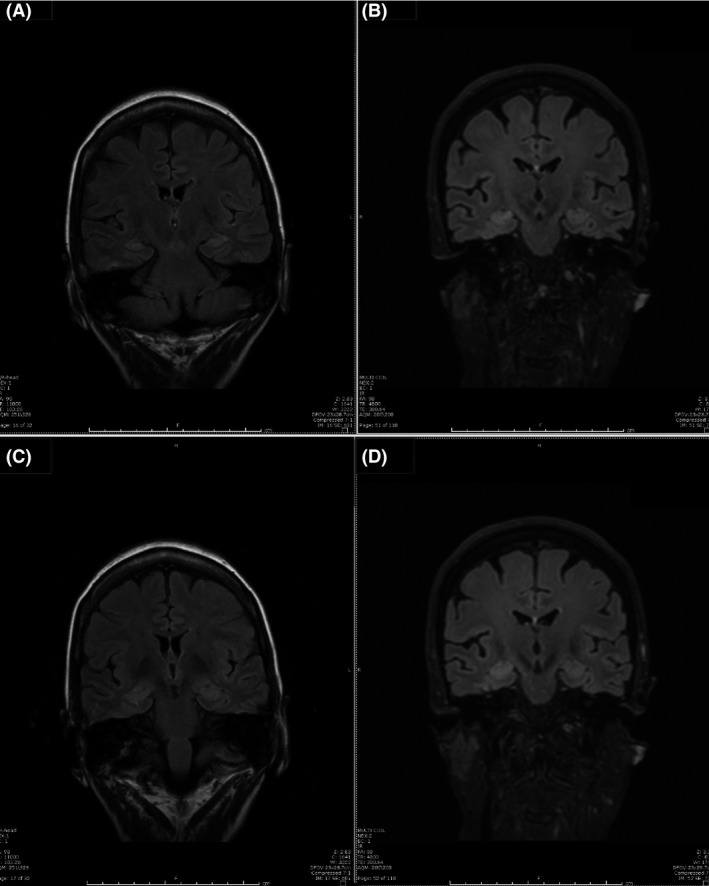
(A and C) MRI of the brain three years prior to video EEG test. B and D: MRI of the brain 4 days after extended video EEG showed subtle increased signal intensity on coronal FLAIR MR sequences in the right hippocampus.

Follow‐up MRI of brain 4 days after this prolonged video EEG showed slightly increased signal abnormalities over right hippocampus on fluid‐attenuated inversion recovery (FLAIR) sequences.(Fig. 5) Compared with MRI of the brain 3 years ago, this recent brain MRI did show progressive atrophy of the brain cortex. However, the bilateral hippocampus remained relatively intact. This slightly increased signal abnormality over right hippocampus could likely represent postictal edema secondary to the clusters of right temporal lobe seizures during this hospital stay.

Prior to this video EEG testing and optimization of antiseizure medication, the patient was deemed a candidate for assisted living by family. Six months after the video EEG, the patient remained independent and asked for the reinstatement of driving privilege. Five minutes delayed recall was 3/3. She was taking levetiracetam 500 mg three times a day and lamotrigine 100 mg twice a day. The benefits of aggressive seizure control on this patient's short‐term memory and management of the activities of daily living were confirmed.

## Discussion

Subclinical seizures denote subtle/clinically unrecognized seizures apparent only on EEG, which indicate focal seizures without impairment of consciousness. There were no observable motor or autonomic components either. Because of epileptic amnesia, patients with subclinical seizures may not be able to recall any subtle semiology of their epileptic seizures.

For patients with subclinical seizures, absence of noticeable clinical presentation, and lack of reliable self‐reported history, subclinical seizure remains to be a challenging diagnosis to make. Clinicians cannot depend on witness statements and self‐reports in this scenario only. EEG tests are necessary.

Positive CSF t‐tau, p‐tau, Aß (_1‐42_), and ATI index were consistent with AD in this case. P‐tau is considered to reflect the phosphorylation state of tau, being a more direct marker for AD. T‐tau reflects the degree of neuronal damage 14. Studies have shown that a combination of these biomarkers can be used to detect AD in subjects with mild cognitive impairment with positive and negative predictive values of >80% 15.

In this case, the epileptic amnesia is probably secondary to prolonged postictal cognitive deficit. Prolonged postictal cognitive deficits could be explained by ongoing ictal activity only recorded on intracranial EEG monitoring but not evident on scalp EEG 16. In this case, abnormal MRI of the brain 4 days after discharge supports the existence of prolonged postictal cognitive deficits.

Dual diagnosis of subclinical seizure and AD remains viable in this case. Specifically, the toxic accumulation of A*β* peptides could trigger abnormal synchronization within the hippocampus, which was reported in human amyloid precursor protein (hAPP) transgenic mice 13, 17, 18.

Single routine scalp EEG is limited in diagnosing subclinical seizures. It is exceptional to be able to record one electrographic seizure like this case in the first outpatient routine scalp EEG. The fact that the first 24 h of inpatient video EEG did not record any electrographic seizure supports the above argument. Spatial sampling in routine scalp EEG is incomplete, as significant amounts of cortex are not covered by standard electrode placement. Seizures deep inside the brain are not evident on scalp EEG. Temporal sampling is also limited, and the relatively short duration of routine interictal EEG recording is another reason why patients with epilepsy may not show interictal epileptiform discharge (IED) in the routine scalp EEG study 19. Therefore, subclinical seizures are likely to be underdiagnosed 3. It is a logical assumption that dementia patients with subclinical seizures would easily go undiagnosed. In a recent study, subclinical epileptiform activity was detected in as many as 42.4% of AD patients 13. More clinical studies are warranted to investigate the percentage of subclinical seizures as the reversible cause of dementia.

The limitations of depending on self‐report alone in providing care to patients with subclinical seizures are evident. Serial or extended EEG would improve the diagnostic yield. Even though these EEG tests are resource intensive, until newer diagnostic tests emerge to replace, serial or extended EEG remains the most reliable investigative tool and should be seriously considered in patients with early‐onset dementia (50–60 years) 13, 17, 18, 20.

## Authorship

ET: assisted in coordinating the patient's care and writing this manuscript. WM: was the neurologist for this patient. YYP: was the neurologist for this patient.

## Conflict of Interest

All authors declare no conflict of interest.
